# 恶性胸腺肿瘤的诊断与治疗进展

**DOI:** 10.3779/j.issn.1009-3419.2010.10.10

**Published:** 2010-10-20

**Authors:** 敬慧 王, 树才 张

**Affiliations:** 101149 北京，北京胸科医院肿瘤内科 Department of Medical Oncology, Beijing Chest Hospital, 101149 Beijing, China

胸腺肿瘤包括来源于胸腺上皮细胞的肿瘤-胸腺瘤和胸腺癌，来源于胸腺淋巴细胞的霍奇金淋巴瘤及其它淋巴瘤，来源于胸腺内分泌细胞的肿瘤-胸腺类癌、燕麦细胞癌等；此外还包括生殖细胞肿瘤、胸腺脂肪瘤、胸腺囊肿、转移癌等。胸腺肿瘤中90%为胸腺瘤，其余是胸腺癌、淋巴瘤及类癌等。胸腺瘤在全部成人恶性肿瘤中不足1%，在成人前纵隔肿瘤中约占30%。据美国国家癌症研究所报告，美国胸腺瘤的发病率为0.15/10万^[1]^，我国尚无相关数据报告。胸腺瘤和胸腺癌的好发年龄为50岁-60岁，中位年龄为56岁，男性发病率高于女性，儿童罕见。胸腺肿瘤的病因尚不明确，有些学者证实部分患者既往有放射治疗、EB病毒感染史。下面将就两种主要的胸腺肿瘤—胸腺瘤和胸腺癌的诊断与治疗等方面的研究进展做一综述。

## 1 病理

WHO胸腺瘤病理分类法是Rosai领导的专家组于1999年公布的，后于2004年得到修改，是目前应用最广泛的胸腺肿瘤病理分类系统。根据肿瘤上皮细胞形态将胸腺瘤分为A型、B型、AB型，A型由梭形肿瘤上皮细胞组成，B型由圆形上皮样细胞组成，具有二者混合表现的为AB型。按照淋巴细胞的比例将B型分为B1型、B2型和B3型3个亚型，B1型富含淋巴细胞，有少量上皮细胞；B2型的淋巴细胞与上皮细胞比例接近；B3型以上皮细胞为主，淋巴细胞稀少。胸腺瘤除了上述分类外，还有几种罕见肿瘤无法归纳至以上标准分类中，包括小结节型胸腺瘤、化生型胸腺瘤、硬化型分类法胸腺瘤等。1999年分类法把胸腺癌定义为C型，2004年删除了胸腺瘤中C型类别，将胸腺癌另列为一类，胸腺癌表现为明显的恶性肿瘤细胞学特征，有多种组织学类型，包括低度和高度恶性肿瘤，低度恶性胸腺癌包括鳞状细胞癌、粘液表皮样癌和基底细胞癌等，高度恶性胸腺癌包括淋巴上皮瘤样癌、小细胞癌、肉瘤样癌、透明细胞癌、未分化肿瘤等。

A型胸腺瘤占5%-24%，患者就诊时年龄大，中位年龄约为61岁，24%合并重症肌无力，绝大多数患者为Ⅰ期。A型和AB型的生物学行为与良性疾病相似，AB型占11%-43%。B型常合并重症肌无力和其它免疫疾病，B1型恶性程度略高于A型、AB型，低于B2型、B3型，是低度恶性肿瘤，占8%-38%。B2型胸腺瘤的恶性程度高于B1型，低于B3型，占4%-46%。B3型以往被定义为分化好的胸腺癌，常无包膜，侵犯纵隔脂肪及邻近器官，预后差，占6%-34%。胸腺癌和其它器官恶性肿瘤相似，复发率高，生存期短，预后最差^[2]^。A型、AB型、B1型、B2型、B3型肿瘤的侵袭性分别为10%-40%、30%-40%、45%-50%、65%-70%、85%-90%，B1型、B2型、B3型侵袭性高，多为Ⅲ期或Ⅳ期疾病^[3]^。

## 2 临床表现

1/3胸腺恶性肿瘤患者表现为无症状的前纵隔肿物，多在影像学检查时发现；1/3表现为局部症状，如：咳嗽、呼吸困难、胸痛、咯血、吞咽困难、声音嘶哑、上腔静脉压迫综合征、膈神经麻痹等；还有1/3患者表现为副瘤综合征，最多见的为重症肌无力，约30%-50%胸腺瘤患者伴重症肌无力，而单纯性红细胞再生障碍、低丙种球蛋白血症、红斑狼疮等约占28%。就诊时胸腺瘤患者出现转移并不常见，最常见的转移部位是胸膜，胸腔外转移不到10%，转移部位有肾、淋巴结、肝、脑、肾上腺、甲状腺、骨。与胸腺瘤不同，胸腺癌侵袭性强，就诊时转移常见，转移部位有骨、肺、肝、胸膜和淋巴结，极少伴有副瘤综合征^[4]^。

## 3 影像学检查

近80%胸腺瘤患者在正位胸片上表现为纵隔轮廓异常或肿物。胸部增强CT是诊断前纵隔肿物的首选影像检查方法，CT不仅能显示病变大小、密度、边缘，而且能提示病变与胸腔内周围器官包括大血管、肺、心包、心脏、胸膜等的关系，肿块内钙化、出血、坏死常提示肿瘤侵袭性高。MRI对评价病变是否侵犯血管更有优势。Sadohara等^[5]^回顾性分析了胸腺肿瘤的CT与MRI的表现，在CT和MRI影像上，与低危(A型、AB型、B1型)和高危(B2型、B3型)胸腺瘤相比，胸腺癌多表现为不规则轮廓、坏死或囊变、不均匀强化、淋巴结肿大及大血管受侵。MRI影像上，与高危胸腺瘤和胸腺癌相比，包膜完整、分隔、均匀强化在低危胸腺瘤更常见，MRI在显示包膜、分隔或瘤内出血方面优于CT。

尽管探讨正电子发射计算机断层扫描(positron emission tomography, PET)对胸腺肿瘤诊断价值的研究多是小样本的，但多数研究显示PET能够区分胸腺增生和胸腺瘤，也可区分胸腺瘤和胸腺癌，胸腺癌的标准吸收值明显高于胸腺瘤。PET摄取值与WHO分类相关，但不能鉴别侵袭或非侵袭性胸腺瘤，也不能鉴别胸腺瘤和淋巴瘤^[6, 7]^。Inoue等^[8]^对46例胸腺肿瘤进行了PET检查，结果显示，高危肿瘤的早期和延迟SUV值(分别为6.0、7.4)均显著高于低危肿瘤(分别为3.2、3.4)。早期SUV值> 4.5支持高危肿瘤，以SUV值4.5为截断值，诊断的敏感性、特异性和准确性分别为78.3%、91.3%、84.8%，早期SUV值> 7.1，可区分胸腺癌与其它类型胸腺瘤。

## 4 诊断

前纵隔肿物的常见病因包括胸腺瘤、淋巴瘤、甲状旁腺或甲状腺肿瘤、生殖细胞肿瘤、结缔组织肿瘤及良性肿瘤等。通过血α甲胎蛋白、β人绒毛膜促性腺激素等标志物可鉴别生殖细胞肿瘤和胸腺瘤。临床上，多数情况下需要对淋巴瘤和胸腺瘤进行鉴别，但这两种疾病鉴别起来较为困难。一般来说，胸腺瘤患者年龄更大，合并相关免疫异常提示为胸腺瘤；淋巴瘤患者多表现盗汗、发热、消瘦和不适等症状，体检时应仔细检查浅表淋巴结，肿块伴周围淋巴结肿大在淋巴瘤多见，可行淋巴结活检确诊。

当发现前纵隔肿物且怀疑是淋巴瘤或胸腺瘤时，应行活检以明确诊断。创伤最小的是CT引导下细针抽吸活检(fine needle aspiration, FNA)，但这种方法只能提供细胞学标本，难以明确胸腺肿瘤的组织学分型，更不能鉴别胸腺瘤和淋巴瘤，因此，FNA一般不用于胸腺肿瘤的诊断。CT引导下芯针活检是确诊纵隔肿物的首选方法，它能获取足够的组织进行组织学及免疫组化检查，从而做出准确诊断，尤其是可鉴别胸腺瘤和淋巴瘤，也比较安全。如果不能进行芯针活检或未确诊时，可考虑行纵隔切开术或电视辅助胸腔镜手术(video-assisted thoracic surgery, VATS)活检。

Yonemori等^[9]^回顾性分析了CT引导下经皮穿刺对138例前纵隔肿瘤的结果，评价其对胸腺上皮肿瘤的诊断价值，敏感性和特异性分别为91.7%和100%，阳性预测值100%，阴性预测值94.0%，总准确率96.4%，经皮穿刺和手术在WHO分类方面的诊断总符合率为79.4%，结果令人满意。另有几项研究报告经皮穿刺的敏感性在44%-83%之间。

## 5 分期

目前采用最广泛的胸腺肿瘤分期方法是Masaoka于1981年制定的Masaoka分期法，后于1994年得到修改。修改后的胸腺瘤Masaoka分期系统的定义是：Ⅰ期：肿瘤局限在胸腺内，肉眼及镜下均无包膜浸润；Ⅱ期：Ⅱa期：镜下有包膜浸润，Ⅱb期：肉眼可见周围脂肪组织浸润，但局限于纵隔胸膜内；Ⅲ期：侵犯周围器官，Ⅲa期不侵犯大血管，Ⅲb期侵犯大血管；Ⅳ期：Ⅳa期：胸膜或心包浸润，Ⅳb期：淋巴或血行转移。Ⅰ期为非侵袭性胸腺瘤，Ⅱ期及以上为侵袭性胸腺瘤。

## 6 治疗

手术、放疗和化疗是胸腺肿瘤主要的3种治疗方法，胸腺癌的治疗与胸腺瘤相似。

### 6.1 手术

手术是治疗胸腺肿瘤的基石，是最有效的治疗方法，所有可能切除的病例均应得到专业团队的仔细评估。对于可切除的前纵隔胸腺瘤，应立即行手术切除。外科切除至少有10种不同的方式，如：经颈部、经胸骨、VATS及联合术式等，但是由于缺乏前瞻性研究结果尚不能证实哪种方式更好。手术第一步应仔细检查纵隔和胸膜腔，肉眼评估有无包膜受侵、肿瘤周围和胸膜受侵程度及周围组织是否受累，注意仔细探查纵隔胸膜，尤其是后横膈窦处有无转移。切除范围包括全部胸腺和周围纵隔脂肪，手术完全切除是治愈最重要的因素。近来研究^[10]^报告手术死亡率不超过2%。

Ⅰ期患者手术切除率为95%-100%，Ⅱ期疾病切除也较为容易，完全切除率为85%-100%，其中分类为B2型、B3型和胸腺癌的患者复发率高。Ⅲ期患者完全切除率为65%-80%，即使术后接受放疗，将近50%患者5年内会复发，复发多在胸膜或肺内，极少扩散至胸腔外。单纯手术不是Ⅳa期胸腺瘤患者的最有效治疗，完全切除率仅为30%-40%。近年来的扩大手术范围治疗及多学科治疗模式明显提高了Ⅳa期患者的生存率，目前资料显示Ⅳa期患者的5年生存率为40%-78%^[11]^。一项大样本的回顾性研究^[12]^发现，切除不完全的胸腺癌患者未能从手术获益。

### 6.2 放疗

胸腺瘤对放疗敏感，放疗在胸腺瘤治疗中起着重要作用，包括术后辅助治疗、局部晚期、不可切除及复发疾病等的治疗。

Ⅰ期胸腺瘤完全切除后复发率极低(0.9%)，术后无需放疗。Ⅱ期胸腺瘤有肉眼包膜、纵隔周围脂肪侵犯，或胸膜粘连，术后复发危险增加，Ⅱ期5年生存率为98%，复发率为4%。对于Ⅱ期患者术后是否行常规放疗曾存在争议，迄今为止，越来越多的研究^[13-16]^证实完全切除的Ⅱ期胸腺瘤患者不会从放疗中获益。而Ⅲ期、Ⅳ期胸腺瘤及胸腺癌术后复发率高(28%, 34%)，术后应行放疗以控制局部复发。

肿瘤分期是决定术后是否放疗的主要依据，但还应考虑肿瘤的WHO类型。对于手术边缘不足够大的Ⅰ期胸腺瘤不推荐放疗，但如果为B3型，则应行放疗。对于Ⅱ期患者，B型患者的复发率高于A型，B2型、B3型和胸腺癌即使无胸膜的镜下侵犯，也会从辅助放疗中受益^[17, 18]^，这些患者放疗和不放疗的纵隔复发率分别为0%、36.4%。Utsumi等^[19]^回顾性分析了324例完全切除的胸腺瘤患者预后，单纯手术切除后的A型、AB型、B1型患者不需放疗，B2型、B3型患者的最佳治疗策略尚需探索。如果病灶紧贴邻近器官，建议行放疗以降低局部复发风险。对于切除不完全的Ⅱ期、Ⅲ期患者应行术后放疗，控制病灶，降低复发率。

仅有几项小型的研究评价了新辅助放疗的作用，证实新辅助放疗可使病灶缩小，肿瘤分期降低，从而提高完全切除率，尤其是对局部晚期患者，但尚无数据显示这种方法对患者长期生存期的影响，因此，新辅助放疗的作用仍存在争议。

一般来说，术后胸腺瘤放疗的推荐剂量为45 Gy-55 Gy。对于术后残存病灶，剂量可达60 Gy。目前推荐的放疗方法有：三维适形放疗或调强放疗，临床靶区包括整个胸腺体积、肿瘤部位、前、上、中纵隔(放疗量至50 Gy-55 Gy时缩野)，不推荐预防性锁骨上淋巴结放疗。胸腺肿瘤放疗的副反应包括急性心包炎、肺炎，晚期副反应有冠脉疾病及肺纤维化^[11]^，近来研究^[20]^报告3度-4度的放疗毒副反应发生率为5%-10%。胸腺癌的放疗剂量尚无一致的结论，多数研究采用的放疗剂量在40 Gy-70 Gy，1.8 Gy/次-2.0 Gy/次。

术后胸膜腔复发是最常见的，因此仅行纵隔放疗不全面。为减少胸膜复发，有研究在纵隔放疗基础上加整个单侧胸腔放疗(剂量15 Gy)，5年无复发率和生存率分别为100%、96%，显著高于仅行纵隔放疗患者(74%, 66%)(*P*=0.03)，但15%-25%患者出现2度或以上的放射性肺炎，肺毒性明显增加，单侧胸腔放疗可能无法成为常规^[21, 22]^。

### 6.3 化疗

化疗可用于晚期胸腺肿瘤的姑息治疗、新辅助化疗及复发疾病的治疗等。在与放疗联合时，顺序为序贯化放疗，以免增加治疗的毒副反应。

胸腺瘤对化疗相对敏感，目前胸腺肿瘤的标准方案是以顺铂、蒽环类为基础的联合方案，有PAC、ADOC、PE、VIP等，但哪种化疗方案最佳尚未明确。表 1、表 2介绍了胸腺肿瘤的几种化疗方案。

**1:** 几项胸腺瘤化疗的试验结果 Results of several trials about chemotherapy efficacy in thymoma

Author	Regimen	Number of patients (*n*)	Response rate (%)	MST(year)
Loehrer^[23]^	PAC (cisplatin 50mg/m^2^, d1; adriamycin 50mg/m^2^, d1; cyclophosphamide 500 mg/m^2^, d1; q3 weeks)	30	50	3.2
Fornasiero^[24]^	ADOC (cisplatin 50 mg/m^2^, d1; adriamycin 40 mg/m^2^, d1; vincristine 0.6 mg/m^2^, d3; cyclophosphamide 700 mg/m^2^, d4; q4 weeks)	32	92	1.25
Giacone^[25]^	PE (cisplatin 60 mg/m^2^, d1; etoposide 120 mg/m^2^, d1-d3; q3 weeks)	16	56	4.3
Loehrer^[26]^	VIP (etoposide 75 mg/m^2^, d1-d4; ifosfamide 1.2 g/m^2^, d1-d4; cisplatin 20 mg/m^2^, d1-d4; q3 weeks)	28	32	2.7
Lemma^[27]^	PC (paclitaxel 225 mg/m^2^, carboplatin AUC 5, q3 weeks)	24	33	> 5
Yokoi^[28]^	CAMP (cisplatin 20 mg/m^2^, d1-d4; adriamycin 40 mg/m^2^, d1; methylprednisolone 1 000 mg/d, d1-d4, 500 mg/d, d5-d6; q3-4 weeks)	14	93	NR
PAC: cisplatin+adriamycin+cyclophosphamide; ADOC: cisplatin+adriamycin+vincristine+cyclophosphamide; PE: cisplatin+etoposide; VIP: ifosfamide+etoposide+cisplatin; CAMP: cispaltin+adriamycin+methylprednisolone; MST: median survival time; NR: not report.				

**2:** 几项胸腺癌化疗的试验结果 Results of several trials about chemotherapy efficacy in thymic carcinoma

Author	Regimen	Number of patients (*n*)	Response rate (%)	MST(year)
Koizumi^[29]^	ADOC (cisplatin 50 mg/m^2^, d1; adriamycin 40 mg/m^2^, d1; vincristine 0.6 mg/m^2^, d3; cyclophosphamide 700 mg/m^2^, d4; q3-4 weeks)	8	75	1.6
Yoh^[30]^	CODE (cisplatin 25 mg/m^2^, weekly; vincristine 1 mg/m^2^, weeks 1, 2, 4, 6, 8; doxorubicin 40 mg/m^2^, weeks 1, 3, 5, 7, 9; etoposide 80 mg/m^2^, for 3 days during weeks 1, 3, 5, 7, 9)	12	42	3.8
Maruyama^[31]^	PC (paclitaxel 200 mg/m^2^, carboplatin AUC6, q3 weeks)	6	100	NR
Lemma^[27]^	PC (paclitaxel 225 mg/m^2^, carboplatin AUC5, q3 weeks)	21	24	1.25
Lgawa^[32]^	PC (paclitaxel 200 mg/m^2^, carboplatin AUC6, q3 weeks)	11	36	1.8
ADOC: cisplatin+adriamycin+vincristine+cyclophosphamide; CODE: cisplatin+vincristine+adrinmycin+etoposide; PC: paclitaxel+carboplatin; MST: median survival time; NR: no report.				

### 6.4 综合治疗

综合治疗包括化疗、放疗、手术3种方法中2种或3种的联合。临床实践证实，对于不可切除疾病，放化疗联合优于单一治疗，对残存疾病或切除不完全的疾病也应考虑多学科综合治疗。对于局部晚期患者，尤其是Ⅲ期患者，术前化疗后行手术、术后行放疗或化疗是可行的，多学科治疗能够提高Ⅲ期或Ⅳa期胸腺瘤患者的手术切除率和生存期。Venuta等^[33]^实施的一项前瞻性新辅助化疗的临床试验结果显示多学科治疗可提高Ⅲ期患者的远期生存率，降低复发率。新辅助化疗的推荐方案为含顺铂的联合方案，术前治疗已成为局部晚期患者治疗方案中重要的组成部分。一项多中心Ⅱ期研究^[34]^正在评价新辅助同步化放疗后手术对Ⅲ期或Ⅳa期胸腺肿瘤患者的治疗效果。

### 6.5 靶向治疗

当前，肿瘤的靶向治疗及其靶点的研究是全球的热点，非小细胞肺癌、乳腺癌、结肠癌等肿瘤的靶向治疗已取得了较好的临床疗效，同时与靶向治疗相关的生物预测因素也日益明确。近来一些研究^[35-39]^报告了胸腺肿瘤EGFR及c-KIT的表达及突变情况，胸腺瘤多表达EGFR而几乎不表达c-Kit，EGFR表达率为71%-94%；胸腺癌则相反，EGFR表达率低于胸腺瘤，为33.3%-67%，c-Kit表达率高，为53%-88%，胸腺瘤和胸腺癌EGFR和Kit的突变罕见。

Kurup等^[40]^采用吉非替尼治疗26例既往接受过治疗的胸腺肿瘤患者(19例转移性胸腺瘤，7例胸腺癌)，250 mg/d，1例部分缓解(partial response, PR)，14例疾病进展(progressive disease, PD)，故作者认为吉非替尼对胸腺肿瘤无效。另一项Bedano等^[41]^报告的Ⅱ期研究采用厄洛替尼联合贝伐珠单抗治疗18例复发的胸腺瘤和胸腺癌，厄洛替尼150 mg/d，贝伐珠单抗15 mg/kg，每3周重复，11例疾病稳定(stable disease, SD)(60%)，7例PD(40%)，厄洛替尼联合贝伐单抗对胸腺肿瘤的疗效也是有限的。Palmieri等^[42]^报告西妥昔单抗治疗2例胸腺瘤的个案报告，2例均获得PR，免疫组化检测EGFR均为阳性。Giaccone等^[43]^采用伊马替尼600 mg/d治疗7例胸腺肿瘤患者(B3型2例，胸腺癌5例)，结果显示，2例SD，5例PD，中位进展时间为2个月，中位生存期4个月，标本检测未发现c-KIT突变。Bisagni^[44]^报告1例经多线化疗的晚期胸腺癌患者接受索拉非尼治疗获得PR，标本经PCR测序显示c-KIT外显子17突变。Strobel等^[45]^报告有KIT强表达及KIT外显子11基因活性突变的1例胸腺癌患者持续6个月对伊马替尼有效。

尽管胸腺肿瘤存在EGFR和c-KIT过度表达，但已有的临床试验结果表明靶向治疗的疗效令人失望，胸腺肿瘤的靶向治疗及预测因素等还需进一步探索。

## 7 复发疾病的治疗

胸腺瘤最常见的复发部位是胸腔，其次是纵隔。对于复发的胸腺瘤患者，手术仍然是主要的治疗方法。50 % - 75 %的复发疾病患者仍可以手术，其中6 2 % (45%-71%)可行完全切除。复发后行完全切除患者的生存期能够得到延长，5年生存率近72%，不完全切除的患者仅为0-25%^[11]^。对于不能手术的患者，放疗是有效的治疗方法，有研究报告接受放疗患者的5年生存率为80%。复发患者也可行化疗，一项评价培美曲塞治疗复发胸腺瘤或胸腺癌的Ⅱ期研究中，共16例复发胸腺瘤和11例胸腺癌入组，在可评价的23例患者中，2例完全缓解(complete response, CR)，2例PR，均为Ⅳa期胸腺瘤，胸腺瘤患者的中位进展时间是45.4周，胸腺癌患者是5.1周，表明培美曲塞对复发胸腺瘤有一定的疗效^[46]^。Loehrer等^[47]^采用奥曲肽治疗奥曲肽扫描阳性的不可切除或晚期胸腺瘤或胸腺癌患者，在可评价的38例患者中，31例曾接受过化疗，结果显示，2例CR，10例PR，2年生存率为76%，6例胸腺癌或类癌患者无PR。另外文献报告紫杉醇、多西紫杉醇、吉西他滨对复发恶性胸腺肿瘤也有一定的效果^[48-50]^。

## 8 预后因素

多种因素能够预测胸腺瘤患者的预后。多项研究证实Masaoka分期是胸腺瘤最重要的预后因素。完全切除患者的生存期与分期密切相关，Ⅰ期、Ⅱ期、Ⅲ期、Ⅳ期的5年生存率分别为90%-100%、75%-90%、50%-70%、30%-40%，Ⅰ期、Ⅱ期、Ⅲ期、Ⅳ期的10年生存率分别为85%-95%、70%-85%、25%-60%、0-15%^[3]^。完全切除是另一个预后因素，完全切除的Ⅲ期、Ⅳ期患者的复发率较未完全切除的复发率低。WHO分类也是手术切除后患者独立的预后因素，从A型到C型(胸腺癌)，预后越来越差。大样本研究及*meta*分析^[51, 52]^均证实，A型、AB型、B1型生存率高于B2型和B3型，有明显差异，A型、AB型、B1型、B2型、B3型的5年生存率分别为100%、93%、89%、82%、71%，A型、AB型、B1型、B2型、B3型的10年生存率约为20%-30%^[3]^。Okumura^[53]^对273例胸腺瘤的研究显示，A型、AB型、B1型、B2型、B3型患者的20年生存率分别为100%、87%、91%、59%、36%。肿瘤大小也是可靠的预后因素，一项对马萨诸塞州总医院179例患者的回顾性分析表明，病灶≥8 cm患者的复发率显著增加(分别为1.8%、28%)，这些患者更多为Ⅲ期或Ⅳ期，多为B1型而不是A型或AB型。大血管受侵也是独立的预后不良因素。此外，伴重症肌无力患者的预后优于不伴肌无力的患者，这可能与合并肌无力患者能更早地就诊有关，而且大多数研究显示，合并重症肌无力的大部分患者是Ⅰ期、Ⅱ期。单纯性红细胞再生障碍(5%-10%)可能影响治疗的完成，尤其是对化疗影响较大。早期复发(< 40个月)也是预后不良因素^[2]^。

## 9 小结

尽管胸腺肿瘤发展相对缓慢，但仍是一种具有侵袭性的疾病。Ⅰ期、Ⅱ期胸腺瘤应行手术切除，部分Ⅱ期患者可从术后放疗获益。多学科综合治疗在Ⅲ期、Ⅳa期胸腺肿瘤中起重要作用。现有的多种化疗方案中大多数包括顺铂和蒽环类药物。靶向治疗尚无令人满意的疗效。今后应针对胸腺肿瘤开展多中心前瞻性临床研究，探索更有效的治疗方法。
